# Modelling Population-Level Hes1 Dynamics: Insights from a Multi-framework Approach

**DOI:** 10.1007/s11538-025-01447-9

**Published:** 2025-05-16

**Authors:** Gesina Menz, Stefan Engblom

**Affiliations:** 1 Division of Scientific Computing, Department of Information Technology, Uppsala University, 751 05 Uppsala, Sweden; 2 Science for Life Laboratory, Department of Information Technology, Uppsala University, Uppsala, Sweden

**Keywords:** Fate decision, Neurogenesis, Cellular synchronisation, Genetic oscillator, Pattern formation, Primary: 92-10, 92B25, 92C15, Secondary: 34A33, 60J20, 34C60, 34F10.

## Abstract

Mathematical models of living cells have been successively refined with advancements in experimental techniques. A main concern is striking a balance between modelling power and the tractability of the associated mathematical analysis. In this work we model the dynamics for the transcription factor Hairy and enhancer of split-1 (Hes1), whose expression oscillates during neural development, and which critically enables stable fate decision in the embryonic brain. We design, parametrise, and analyse a detailed spatial model using ordinary differential equations (ODEs) over a grid capturing both transient oscillatory behaviour and fate decision on a population-level. We also investigate the relationship between this ODE model and a more realistic grid-based model involving intrinsic noise using mostly directly biologically motivated parameters. While we focus specifically on Hes1 in neural development, the approach of linking deterministic and stochastic grid-based models shows promise in modelling various biological processes taking place in a cell population. In this context, our work stresses the importance of the interpretability of complex computational models into a framework which is amenable to mathematical analysis.

## Introduction

The Hes1 protein is part of a family of helix-loop-helix repressors which sustain progenitor cells during development and induce binary cell differentiation processes (Kageyama et al. 2007). Hes1, specifically, plays an important role during neuronal development and the development of parts of the digestive tract during embryogenesis, as well as being found to contribute in tumours by ways of maintaining cancer stem cells and aiding metastasis (Shimojo et al. 2011; Kageyama et al. 2007; Liu et al. 2015). The exact molecular interactions of these processes, however, are not yet entirely understood (Kobayashi and Kageyama 2014), making Hes1 interesting for mathematical modelling purposes to investigate potential interactions.

To maintain neural progenitor cells, Hes1 oscillates due to a negative feedback loop between the Hes1 protein and the Hes1 gene (Hirata et al. 2002; Shimojo et al. 2011). Interactions between the Hes1 negative feedback loop with the Delta-Notch pathway, a well-conserved developmental pathway influencing organ development have, in particular, been observed in developing neural tissue of model organisms such as mice (Imayoshi et al. 2013; Shimojo et al. 2016) and with the Hes1 homologue Her6 in zebrafish (Soto et al. 2020). These interactions then lead to oscillations throughout a cell population for a few cycles which dampen over time (Shimojo et al. 2008; Phillips et al. 2016), finally resulting in a sustained “salt and pepper pattern” of cells with high and low levels of Hes1 throughout the population (Imayoshi et al. 2013; Artavanis-Tsakonas et al. 1999; Kageyama et al. 2007). Within this pattern, cells with low Hes1 levels differentiate into neurons via lateral inhibitions while cells with high levels of Hes1 become supporting glial cells as observed during, e.g., mouse brain development (Imayoshi et al. 2013; Kageyama et al. 2008). To allow for the development of sufficient numbers of each cell type, progenitor cells need to be maintained at appropriate levels (Shimojo et al. 2011). Although originally believed to act like a molecular clock similar to the cell cycle, more recent research suggests that Hes1 oscillations do not specifically time neural development during embryogenesis but rather allow cells to stay undifferentiated for a sufficient amount of time before differentiation to allow appropriate tissue composition (Hirata et al. 2002; Kobayashi and Kageyama 2014). In this context, however, all details and functions of Hes1 behaviour have not yet been understood leading to various mathematical models seeking to understand and/or explain aspects of these highly complex molecular interactions. We next review a few modelling frameworks that have been proposed for the Hes1 system.

One type of model that has been explored multiple times is a relatively simple ordinary differential equation (ODE) model in a single cell aimed purely at understanding how oscillations can occur via a negative feedback loop such as in the Hes1 system. Such work has been done by investigating how Hes1 protein, Hes1 mRNA and an intermediary factor interact (Hirata et al. 2002), what role *delay* plays in establishing oscillations (Barrio et al. 2006; Monk 2003; Jensen et al. 2003; Momiji and Monk 2008), as well as the function of dimerisation of the Hes1 protein before it attaches to the Hes1 promoter (Zeiser et al. 2007), showing that each of these models can generate sustained oscillations.

Single cell models have been extended to include more detailed ODE and partial differential equation (PDE) descriptions. These models account for interactions between the Hes1 negative feedback loop and other cellular pathways, such as the cell cycle (Pfeuty 2015), the cell-internal dynamics including the accumulation of the microRNA miR-9 (Goodfellow et al. 2014; Phillips et al. 2016) and the Notch pathway (Pfeuty 2022; Tiedemann 2017), as well as the spatial distribution of components throughout the cell (Agrawal et al. 2009). These refinements preserve oscillatory behaviour while, under specific conditions, allowing for stable pattern formation in a cell population.

Additionally, the gene regulatory networks (GRNs) of both the Hes1 and the related Hes5 proteins have been explored using various stochastic modelling approaches. The Hes1 GRN, in particular, has been effectively captured using the reaction-diffusion master equation (RDME) approach. By modelling the Hes1 signalling pathway within a single cell represented by a computational mesh, the RDME method provides a detailed view of the system by modelling gene regulatory behaviour on a single interaction basis. This approach not only captures oscillatory dynamics even in the presence of noise but also allows for deeper investigation into how nuclear transport and dimerisation influence the system (Sturrock et al. 2013, 2014). Similarly, both the Hes1 and the Hes5 GRN have been modelled using both delay stochastic models such as delay stochastic simulation algorithm models (Barrio et al. 2006), chemical master equation (Phillips et al. 2016) and stochastic differential equation methods (Manning et al. 2019; Hawley et al. 2022), highlighting the versatility of stochastic approaches in capturing the complex dynamics of these networks as well as examining how delays contribute to typical oscillatory behaviour in both single cell (Agrawal et al. 2009; Sturrock et al. 2014; Barrio et al. 2006; Phillips et al. 2016; Goodfellow et al. 2014; Manning et al. 2019) and multicellular (Pfeuty 2022; Tiedemann 2017; Hawley et al. 2022) environments. Although these models describe the Hes1, and related Hes5, pathway in greater detail, they are also increasingly complex, making it hard to understand their behaviour analytically.

Zooming out from the Hes1 specifics and focusing mainly on developmental patterning in general, the Delta-Notch pathway has been modelled in multiple ways: From very basic models to determine patterning behaviour while remaining conducive to analysis (Collier et al. 1996), to further extensions including protrusions and, thus, inducing more extensive patterns than salt and pepper patterns (Cohen et al. 2010; Sprinzak et al. 2011; Hadjivasiliou et al. 2016; Engblom 2019). In the two-cell and one-dimensional case, oscillatory behaviour followed by stable patterning has also been found by including delay into the Delta-Notch system (Veflingstad et al. 2005; Momiji and Monk 2009). Investigations of travelling wavefronts within neurogenesis and the influence of cell morphology on patterning behaviour (Formosa-Jordan 2012; Saleh et al. 2021) have shown that patterning is stable across different environments. While some previous models explicitly include the Hes1-Notch connection (Agrawal et al. 2009; Pfeuty 2015), models purely focusing on the Delta-Notch pathway are also interesting to us since they have formalised the description of Delta-Notch behaviour and are amenable to mathematical analysis thanks to a lower model complexity (Collier et al. 1996).

We aim at investigating models across different frameworks and start by modelling the underlying GRN using an ODE system on a grid, based on the schematics of the biological process. For this we use parameters drawn from the literature and otherwise determined to the best of our knowledge. This model captures the oscillatory behaviour followed by stable expression of Hes1 which indicates fate decision into either neurons (at low levels of Hes1) or glial cells (at high levels) while keeping the number of modelled molecular regulators to a minimum. However, this system is still fairly complex and difficult to analyse so we reduce it to a two-dimensional and even scalar ODE using quasi-steady state assumptions. In this way, we find four apparently different but closely related reduced systems which, although they do not capture the oscillations, allow us to analyse the timing and behaviour of the fate decision process. We further extend our ODE model to a spatial stochastic RDME model (Engblom et al. 2009; Drawert et al. 2012) to be able to experience with the system’s stability to intrinsic noise.

We have structured the paper as follows. In Sect. 2 we detail the Hes1-Notch signalling model under consideration. Sources of stochasticity from intrinsic cellular noise as well as spatial effects are included. We analyse the spectral properties of the model in Sect. 3, first assuming a deterministic framework and using linear stability analysis in space. We investigate the precision of the analysis as well as its relevance for a more realistic spatially extended stochastic model. A concluding discussion around the themes of the paper is found in Sect. 4.

## Models

Both Hes1 and the Notch pathway are well-preserved pathways and important during embryonic development (Chen et al. 1997; Pompa et al. 1997) As a fundamental pathway within neurogenesis, it has been extensively analysed through experiments but a single description of a GRN within one cell or even the small neighbourhood of its immediate surrounding cells found from such experiments does not lead into insights into how single cell interactions lead to population-level behaviour. This motivates our interest in modelling this behaviour mathematically.

In this section we first describe the underlying biology of the combined Hes1-Notch GRN in Sect. 2.1. Next, we present an ODE interpretation of this biological pathway on a population of cells in Sect. 2.2. Finally, in Sect. 2.3 we set up a stochastic model of the Hes1-Notch pathway, again on the cell population level, but using the Reaction-Diffusion Master Equation (RDME) framework.

### Hes1 Cell-to-Cell Signalling Process

For the modelling, we would ideally like to describe the pathway in a way that captures its main behaviours while allowing us insight into mechanistic interactions on a population level through mathematical analysis and computational simulations. The behaviour we want to capture is the behaviour shown in neural progenitor populations where Hes1 shows transient oscillations with a period of 2–3 h (Marinopoulou et al. 2021; Imayoshi et al. 2013; el Azhar et al. 2024) sometimes extending up to 4 h (el Azhar et al. 2024). This is then followed by a fate decision into stationarity, with either high or low Hes1 protein levels leading to cells developing into glial cells or neurons, respectively (Shimojo et al. 2008), which from biological considerations has to be rather robust to process and environment noise.

**Fig. 1:**
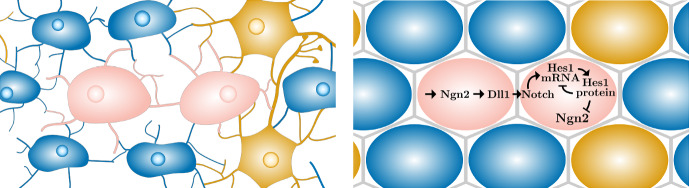
*Left:* Representation of neurons (orange), glial cells (blue) and undifferentiated cells (pink) in a developing brain. *Right:* Schematics of the Hes1 negative feedback loop in two neighbouring cells. The same interactions occur in every cell throughout the neural progenitor cell population between all neighbouring cells. All arrows ending with an arrowhead denote an activation or creation of a constituent while arrows ending with a vertical line denote a repression. All constituents are also degraded (not shown) (Color figure online)

We start with the schematic understanding of the underlying biological processes depicted in Fig. 1. Following (Shimojo et al. 2011), the main molecules involved in the process of maintaining neural progenitor cells are Neurogenin-2 (Ngn2), Delta-like-1 (Dll1), the Notch receptor as well as Hes1 mRNA and protein which together interact as indicated in the figure. To this end we use the notation

1 $$\begin{aligned} D \text {: Dll1, } N \text {: Notch, } M \text {: Hes1 mRNA, } P \text {: Hes1 protein, } n \text {: Ngn2,} \end{aligned}$$

for the concentrations of respective constituents in each cell.

Starting in the left pink cell in Fig. 1, Ngn2 is constitutively produced and induces the production of Dll1, which in turn is presented on the cell membrane and interacts with the Notch receptor on the surface of the right cell. The internal part of the Notch receptor, the Notch intracellular domain, then interacts with the Hes1 gene promoter to induce the production of Hes1 mRNA which we summarise here as Notch inducing Hes1 mRNA production. However, we are aware that both Dll1 and Notch have a bound/inactive form as well as a free/active form. Both proteins are transmembrane molecules and signalling occurs via direct contact between the proteins (Bray 2016). This direct contact renders both proteins unable to function after signalling which becomes relevant during the mathematical modelling process.

Following the biological pathway further, Hes1 protein is then produced from the Hes1 mRNA and successively inhibits the production of new Hes1 mRNA while also repressing the production of the proneural protein Ngn2. Ultimately, this process causes all key components, including Dll1 and Ngn2, to oscillate before stabilising into a “salt and pepper" pattern (Shimojo et al. 2008, 2016; Kageyama et al. 2008). In this pattern, cells with high levels of Hes1 protein are surrounded by cells with low Hes1 levels while cells with low levels of Ngn2 and Dll1 tend to have high Hes1 protein levels, while those with high Ngn2 and Dll1 levels have low Hes1.

### Network ODE Models

Given the schematic understanding of Fig. 1, we start by proposing an ODE model to describe the Hes1-Notch GRN within a single cell. In this case we describe Dll1, Notch, Hes1 mRNA, Hes1 protein and Ngn2 as concentrations [*D*, *N*, *M*, *P*, *n*], hence extending purely Delta-Notch signalling systems such as (Collier et al. 1996; Cohen et al. 2010; Formosa-Jordan 2012) to also include the Hes1 negative-feedback dynamics similar to the model presented in Pfeuty (2022), but without including delay.

We let all molecules be degraded at a rate $\mu _i$ with $i \in \{D,N,M,P,n\}$ and capture the inhibition of Hes1 mRNA as well as the repression of the production of the proneural protein Ngn2 using the repressor form of Hill functions of the Hes1 protein (Alon 2006). At the same time, the activation or production of each constituent is modelled using $\alpha _i$ according to individual dynamics of each molecule. These considerations lead to the system describing the Hes1-Notch GRN in a single cell to be

2 $$\begin{aligned} \left. \begin{array}{rcl} \dot{D} & =& \alpha _D n - \mu _D D, \\ \dot{N} & =& \alpha _N \langle D_{\text {in}}\rangle - \mu _N N, \\ \dot{M} & =& \frac{\alpha _M N}{1 + (P/K_M)^k} - \mu _M M,\\ \dot{P} & =& \alpha _P M - \mu _P P, \\ \dot{n} & =& \frac{\alpha _n}{1+(P/K_n)^h} - \mu _n n. \\ \end{array} \right\} \end{aligned}$$

Here, $\langle D_{\text {in}}\rangle := \sum _i w_i D_i$ is the average time-dependent Dll1 signal a cell receives from its neighbouring cells *i* (always normalising the weights $w_i$ to sum to unity). To determine the cell population behaviour, we apply this ODE system on each individual node in a network which represents the connectivity between a population of cells. In this paper we mainly use regular hexagonal grids, however, other grids can easily be treated in the same way.

**Fig. 2:**
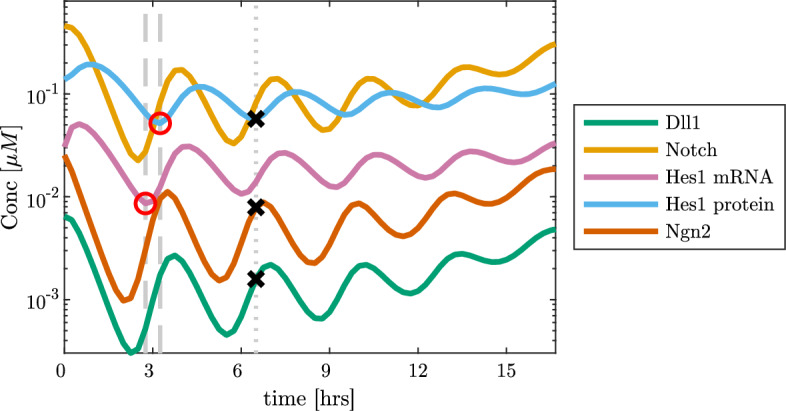
Dynamics averaged over all cells on a 20-by-20 grid of hexagonal cells when starting from random initial data. The two dashed vertical lines indicate the offset between Hes1 mRNA and Hes1 protein expression levels which has been shown previously in Hirata et al. (2002). The offset between the Hes1 mRNA and Hes1 protein oscillations between the two markers as shown is approximately 30 min. The vertical dotted line shows that we approximately capture the inverse oscillations between the Hes1 protein, and Dll1 and Ngn2 (Shimojo et al. 2011) (color figure online)

We propose the parameters as given in Table 1. Since both the timings of the entire process with oscillations of periods $\sim$ 2–3 h (Imayoshi et al. 2013; Marinopoulou et al. 2021), as well as most parameter values are available for mouse embryonal cell lines, our overall calculations are based on these timings for mouse development. For the degradation rates $\mu _i$ we rely on the half-lifes for the associated components except for *D* and *N* which, as previously mentioned in Sect. 2.1, become inactive upon contact made by signalling due to proteolytic cleavage of the Notch receptor. Thus, we assume that $80\%$ of both proteins are used while $20\%$ are free and can be degraded, i.e., that the measured degradation rates are those of the $20\%$ free transmembrane proteins, thus, causing the actual degradation rates to increase fivefold, cf. Table  1. One element deciding system behaviour is the choice of the Hill coefficients *k* and *h*. We require both $k,h \in \mathbb {N}^+$ and choose $k = 1$ and $h = 4$ as these are the minimum values which we have found are necessary to realistically capture oscillations. Similarly, we choose the Hill-function dissociation constants $K_M$ and $K_n$ to match the overall system behaviour, as these primarily influence the oscillation period and number of oscillations. Since the system is underdetermined, we do not account for perturbations in these values. Given degradation rates and with fixed Hill functions, our activation rates $\alpha _i$ follow by fitting to the relative amounts of each component as found in Huang et al. (2023). The uncertainty of these activation rates are found by a straightforward Monte Carlo approach, using the independent perturbations in Table 1 and assuming $5\%$ noise for the concentrations. For more information about this, see Appendix A. The resulting typical dynamics of the model are shown in Fig. 2.

**Table 1:** Parameters for (2)

Parameter	Value (68% confidence interval)	Reference
$\alpha _D$	$0.018 \ (0.016, 0.021)$ [/min]	*This paper*
$\alpha _N$	$6.0 \ (5.3, 6.7)$ [/min]	
$\alpha _M$	$0.017 \ (0.016, 0.019)$ [/min]	
$\alpha _P$	$0.14 \ (0.12, 0.16)$ [/min]	
$\alpha _n$	$0.0049 \ (0.0043, 0.0054)$ [$\upmu$M/min]	
$\mu _D$	$\log 2/50 \times 5 \ \log (2) / (45.3,55.2)\times 5$ [/min]	Dll1 half-life in mice Shimojo et al. (2016)
$\mu _N$	$\log 2/40 \times 5 \ \log (2)/(36.2,44.2) \times 5$ [/min]	Notch1 half-life in humans Agrawal et al. (2009)
$\mu _M$	$\log 2/24.1 \ \log 2 /(22.4, 25.8)$ [/min]	Hes1 protein half-life in mice Hirata et al. (2002)
$\mu _P$	$\log 2/22.3 \ \log 2/(19.2,25.4)$ [/min]	Hes1 mRNA half-life in mice Hirata et al. (2002)
$\mu _n$	$\log 2/21.9 \ \log 2/(19.7, 24.1)$ [/min]	Ngn2 half-life in Xenopus Vosper et al. (2007)
$K_M$	$\equiv 0.050$ [$\mu$ M]	*This paper*
$K_n$	$\equiv 0.030$ [$\mu$ M]	
*k*	$\equiv 1$	*This paper*
*h*	$\equiv 4$	

Values of $\alpha _i$ are chosen to give the desired behaviour of constituents relative to each other (Huang et al. 2023), while $\mu _i$ values are based on the half-lives of the components of the GRN, mostly in mice. For *D* and *N*, specifically, we make the modelling assumption that $80\%$ of each is bound, thus, leading to the multiplication by the factor 5. Where values are available with error estimates we use those, while for $\alpha _i$ we fit them to all other perturbed parameters, and for $\mu _D$ and $\mu _N$ we assume an ad hoc $\pm 10\%$ uncertainty since the value for $\mu _N$ is more uncertain (a higher range of values including an NICD half-life of $\sim 180$ min (Ilagan et al. 2011) has been reported). To achieve the behaviour we desire, the value from Agrawal et al. (2009) was used for this parameter

**Fig. 3:**
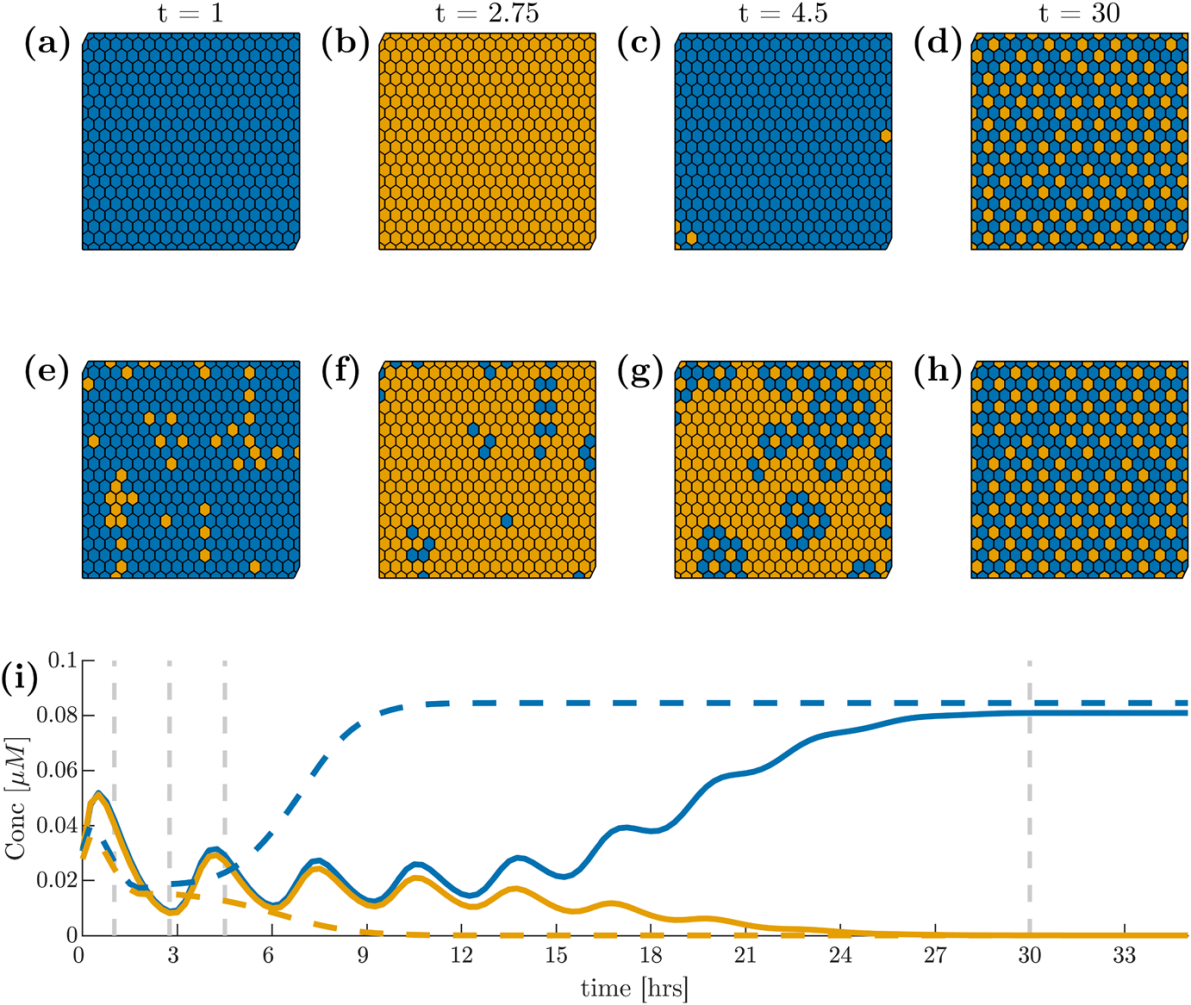
(**a**)–(**d**) Spatial dynamics of Hes1 mRNA in our proposed grid ODE model (2) where blue cells are above the mean concentration before fate decision and orange cells are below this threshold. (**e**)–(**h**) Hes1 mRNA in the reduced model (3) on the same grid. (**i**): the average Hes1 mRNA (solid line: full ODE model; dashed line: reduced model) over time calculated separately over all cells which show high or low Hes1 concentrations after fate decision with blue and orange denoting high and low expression, respectively. The vertical lines denote the times at which the spatial dynamics are shown in the top and middle rows. All simulations shown are on a $20 \times 20$ grid with zero boundary conditions. Initial conditions are uniform random values scaled to the required concentrations as given in Appendix A. Note that by our parameterisation, we find our results in concentrations (Color figure online)

For improved ability to analyse the system, we assume quasi-steady states for three of the five states to find a reduced ODE system. Depending on the reduction we choose, we find either equations of type 1,

3 $$\begin{aligned} \left. \begin{array}{rclcl} \dot{x} & =& \frac{\langle y_{\text {in}}\rangle }{a+x^k} - x & =:& \langle y_{\text {in}}\rangle f(x) - x\\ \dot{y} & =& v \left( \frac{1}{1 + b x^h} - y \right) & =:& v \left( g(x) - y \right) \end{array} \right\} , \end{aligned}$$

or of types 2 and 3, respectively,

4 $$\begin{aligned} \left. \begin{array}{rcl} \dot{x} & =& y f(x) - x \\ \dot{y} & =& v \left( \langle g(x_{\text {in}}) \rangle - y \right) \end{array} \right\} , \qquad \left. \begin{array}{rcl} \dot{x} & =& \langle g(y_{\text {in}}) \rangle f(y) - x \\ \dot{y} & =& v \left( x - y \right) \end{array} \right\} , \end{aligned}$$

where *f* and *g* are as for type 1 and where $\langle g(x_{\text {in}}) \rangle = \sum _i w_i g(x_i)$ is the average of $g(x_i)$ across the neighbour cells *i*. Overall, there are 10 possible ways to reduce the original system (2) to a two-dimensional system by making quasi-steady state assumptions. However, three possible options, those where neither of *x* and *y* corresponds to *M* or *P*, are not readily reducible since the reduction involves solving Hill equations. This leaves seven possible alternatives (four of type 1, two of type 2 and one of type 3) capturing the steady state behaviour of the original system (2). The different alternatives are summarised in Table  2, and a typical derivation can be found in Appendix B. For comparison, the behaviour of both the full model (2) and the best fit reduced model (3) are shown in Fig. 3. To note about the reduced models in (3) and (4) is that they all end up with the same parameters *a* and *b*, cf. Table  2, while *v* varies such that all three reduced model types behave similarly except for the timing of fate decision which is determined by *v*.

To further simplify analysis, at points we use a scalar version of our model. To reach this, we make the further assumption that $\dot{y} = 0$ in either of the two-dimensional models (3)–(4). This reduces all three types into

5 $$\begin{aligned} \dot{x} = \langle g(x_{\text {in}}) \rangle f(x)-x. \end{aligned}$$

Our reduced models (3)–(4) are remindful of the Delta-Notch model from Collier et al. (1996),

6 $$\begin{aligned} \left. \begin{array}{rclcl} \dot{x} & =& \frac{\langle y_{\text {in}} \rangle ^k}{a + \langle y_{\text {in}} \rangle ^k} - x & =:& F(\langle y_{\text {in}} \rangle ) - x\\ \dot{y} & =& v \left( \frac{1}{1 + b x^h} - y \right) & =:& v \left( G(x) - y \right) \end{array} \right\} , \end{aligned}$$

where *x* describes Notch, *y* describes Delta, and $\langle y_{\text {in}}\rangle$ is the average incoming Delta from the neighbours on the grid. While our models (3) and (4) show differences in the form of *f*(*x*), the order of averaging and Hill functions, the values of the Hill coefficients *k* and *h*, as well as where the model links the incoming signal compared to the Collier model (6), we can use an analysis similar to the one proposed in Collier et al. (1996) to investigate the behaviour of our system further.

**Table 2:** The seven alternative ways to reduce the original system (2) to (3) or (4) via quasi-steady state assumptions and the resulting effective parameter *v*

type	*x*	*y*	*v*
**1**	$\textbf{M}$	$\textbf{n}$	$\mathbf {1.096 \ (0.975, 1.280)}$
1	*P*	*n*	$1.014 \ (0.851, 1.100)$
1	*M*	*D*	$2.410 \ (2.111, 2.740)$
1	*P*	*D*	$2.230 \ (1.800, 2.627)$
2	*M*	*N*	$3.013 \ (2.712, 3.401)$
2	*P*	*N*	$2.788 \ (2.298, 3.280)$
3	*M*	*P*	$1.081 \ (0.936, 1.305)$

The parameters *a* and *b* are $0.083 \ (0.071, 0.094)$ and $1.652 \times 10^5 \ (0.807, 3.217) \times 10^5$ for all alternatives ($68\%$ confidence intervals). The version displayed in Fig. 3 is indicated in bold

### Spatial Stochastic Reaction-Transport Model

To take intra-cellular noise into account we also consider a mesoscopic stochastic version of the grid ODE (2) as follows. We represent the individual cells as nodes in a network with connectivity given by an underlying mesh discretisation. Consider a single cell first, with time-dependent state vector $X(t) \in \textbf{Z}_{+}^{d}$ counting at time *t* the number of constituents (or species) in each of *d* compartments. We may generally prescribe *R* Markovian reactions in the form of Poissonian state transitions $X \mapsto X+\mathbb {N}_{r}$ by

7 $$\begin{aligned} \textbf{P}\left[ X(t+dt) = x+\mathbb {N}_{r}| \; X(t) = x\right]&= w_{r}(x) \, dt+o(dt), \end{aligned}$$

for $r = 1\ldots R$ with $w_r(x)$ the *r*th transition intensity (or propensity), and $\mathbb {N}\in \textbf{Z}^{d \times R}$ the stoichiometric matrix. The evolution of the *i*th species can then be described by the Poisson representation (Ethier and Kurtz 1986)

8 $$\begin{aligned} X_i(t)&= X_i(0)+\sum _{r = 1}^{R} \mathbb {N}_{ri} \Pi _{r} \left( \int _{0}^{t} w_{r}(X(s)) \, ds \right) , \end{aligned}$$

with unit-rate and independent Poisson processes $(\Pi _{r})_{r = 1}^{R}$.

**Fig. 4:**
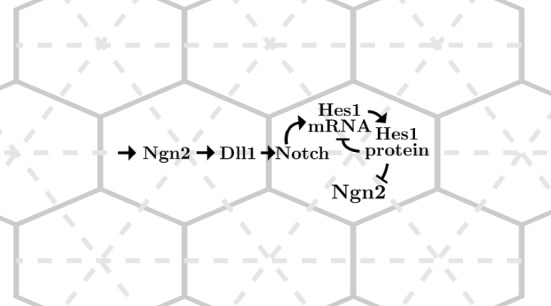
The schematics as implemented on a hexagonal grid (solid lines) using the RDME model. The dashed lines show the triangulation on which the hexagonal grid is built in the URDME framework

In the present case we identify the following reactions:

9 $$\begin{aligned} \left. \begin{array}{rcl} n & \xrightarrow { \alpha _Dn } & n+D \\ N & \xrightarrow { \alpha _M N/(1+(P/(K_M V))^k) } & N+M \\ M & \xrightarrow { \alpha _P M } & M+P \\ \emptyset & \xrightarrow { \alpha _nV/(1+(P/(K_nV))^h) } & n \end{array} \right\} \qquad \left. \begin{array}{rcl} D & \xrightarrow { \mu _D D } & \emptyset \\ N & \xrightarrow { \mu _N N } & \emptyset \\ M & \xrightarrow { \mu _M M } & \emptyset \\ P & \xrightarrow { \mu _P P } & \emptyset \\ n & \xrightarrow { \mu _n n } & \emptyset \end{array} \right\} \end{aligned}$$

where $V$ is the volume of each voxel. The production of Notch, as initiated by the Dll1 signal, is yet to be described.

We next consider a population of cells in *K* nodes or voxels $(V_k)_{k = 1}^K$ and a time-dependent state $X \in \textbf{Z}_{+}^{d \times K}$, with $X_{ik}(t)$ the number of constituents of the *i*th species in the *k*th voxel. The general dynamics (8) now becomes

10 $$\begin{aligned} X_{ik}(t) = X_{ik}(0)&+ \sum _{r = 1}^{R} \mathbb {N}_{ri} \Pi _{rk} \left( \int _{0}^{t} V_k u_{r}(V_k^{-1}X_{\cdot ,k}(s)) \, ds \right) \nonumber \\&-\sum _{k = 1}^{J} \Pi _{ijkl}' \left( \int _{0}^{t} q_{ijkl}X_{ik}(s) \, ds \right) +\sum _{k = 1}^{J} \Pi _{jilk}' \left( \int _{0}^{t} q_{jilk}X_{jl}(s) \, ds \right) , \end{aligned}$$

where $q_{ijkl}$ is the rate per unit of time for species *i* in the *k*th voxel to transfer into species *j* in the *l*th voxel, and where $(\Pi _{\cdot },\Pi '_{\cdot })$ is an appropriately extended set of independent unit-rate Poisson processes. This general linear transfer process is not standard as it allows for species to change their type while transporting, but it is appropriate here since it is exactly this effect we are interested in. Note also that in (10), the propensities $(u_r)$ are independent of the voxel volume $V_k$. Using this formalism we may augment (9) with

11 $$\begin{aligned} \left. \begin{array}{rrl} D & \xrightarrow { \alpha _N D }& D+D^{\text {in}} \\ D_k^{\text {in}} & \xrightarrow { \alpha _N q_{kl} D_k^{\text {in}}}& N_l \end{array} \right\} \end{aligned}$$

that is, a Dll1 signal in voxel *k* sequentially transforms into a diffusing pseudo species $D^{\text {in}}$, which then diffuses *into* a Notch signal in voxel *l* at rate $\alpha _N q_{kl}$, where $q_{kl}$ is the proportion of Dll1 used for the signal between these two voxels (for example, $q_{kl} \equiv 1/6$ on a hexagonal mesh with *k* and *l* neighbouring voxels).

**Fig. 5:**
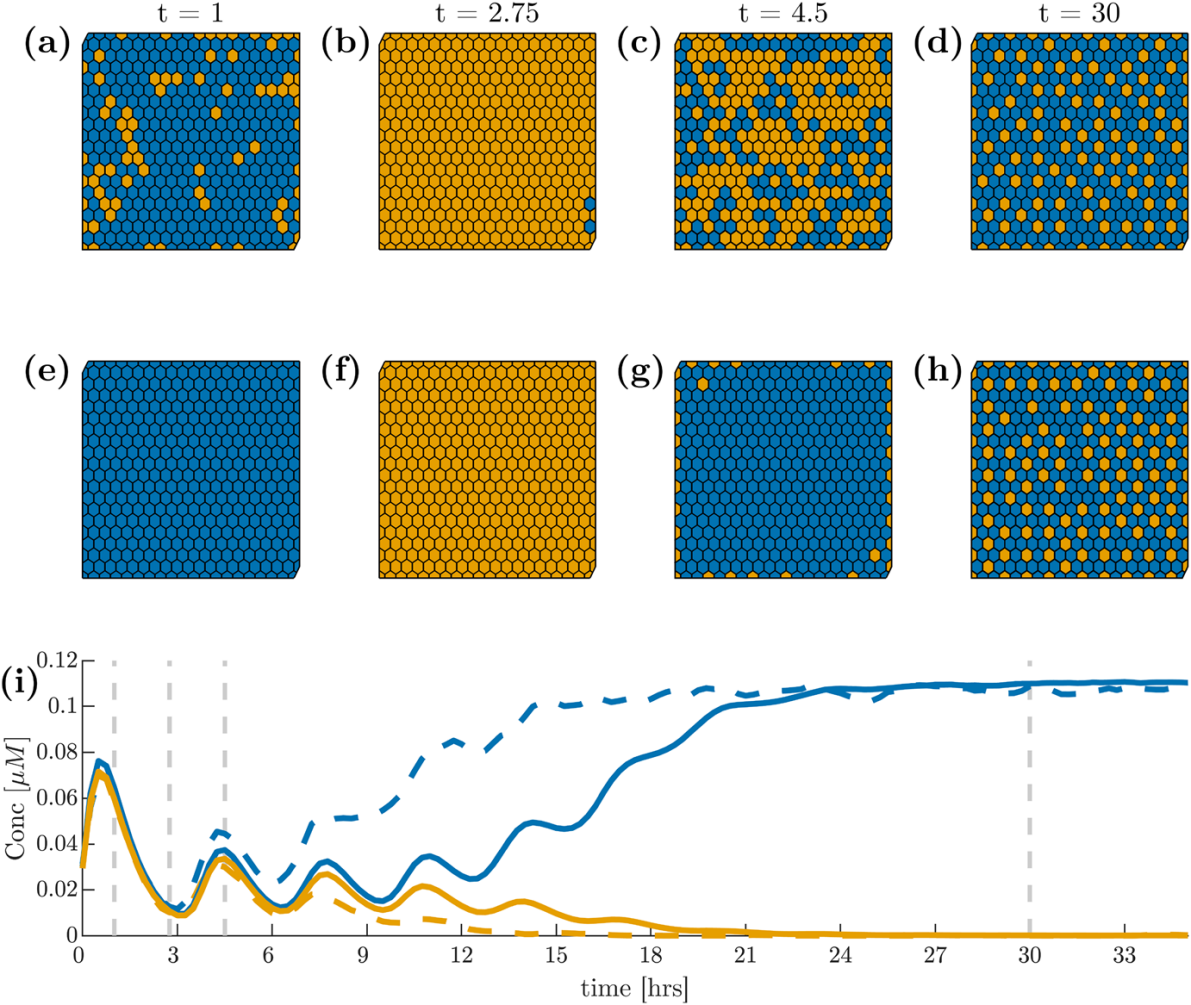
(**a**)–(**d**) spatial dynamics of Hes1 mRNA in our RDME model (9) and (11) choosing the volume of each voxel to be $1\,\upmu$m$^3$, representing a rather high noise levels, and using the same colour scheme as in Fig. 3. (**e**)–(**h**) Hes1 mRNA in the RDME model with voxel volume $50\, \upmu$m$^3$, i.e., less levels of noise. (**i**) the average Hes1 mRNA at low volume, $1\,\upmu$m$^3$, (dashed line) and high volume, $50\,\upmu$m$^3$, (solid line) over time where the horizontal lines denote the times at which the spatial dynamics are shown in the top and middle rows. Blue and orange, again, denote cells with high and low expression and boundary and initial conditions are chosen as previously only this time initial conditions are in number of molecules. Based on a mouse embryonal stem cell volume of approximately $50\,\upmu$m$^3$ (size based on Pillarisetti et al. (2009); Wang et al. (2011) assuming spherical cells) and a mean number of 8104 molecules per cell (Ho et al. 2018), we find our results in $\upmu$M (Color figure online)

The model so described can readily be implemented across a given triangulation of space using URDME (Drawert et al. 2012) and simulated using the supported NSM-solver with a triangulation as illustrated in Fig. 4. Sample simulations are reported in Fig. 5.

## Analysis and Results

We next analyse the properties of the system (2). Existence and qualitative behaviour of fate decision in a two-cell 1D periodic system in the reduced model (3)–(5) is investigated in Sect. 3.1 and in the full model (2) in Sect. 3.2. We then examine the behaviour of the system (3) on a regular hexagonal grid in Sect. 3.3, and we finally quantitatively compare the patterning differences between the ODE (2) and RDME models (9)–(11) in Sect. 3.4.

### The Reduced Stationary Solutions

**Fig. 6:**
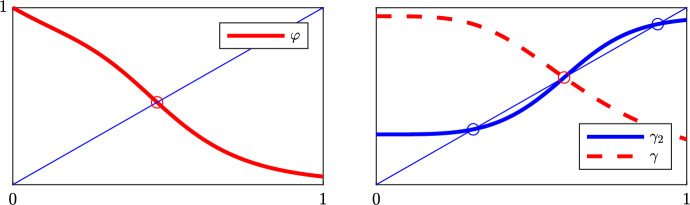
Fix point arguments. *Left:* the unique homogeneous stationary state is the fix point $\bar{x}_0 = \varphi (\bar{x}_0)$. *Right:* if $\gamma _2'(\bar{x}_0) > 1$, then there are cyclic (non-homogeneous) solutions $\bar{x}_1< \bar{x}_0 < \bar{x}_2$ (color figure online)

At stationary solutions to (2), the quasi-stationary arguments used to arrive at the reduced systems (3)–(5) are valid and so we target these models initially. We first consider the homogeneous steady state where, by “homogeneous” we simply mean that all cells have identical states. We pick the scalar reduced model (5), i.e.,

12 $$\begin{aligned} \dot{x}&= g(\langle x_{\text {in}}\rangle ) f(x)-x, \end{aligned}$$

where *f*, *g* are as in (3). Looking for a homogeneous steady state where $\langle x_{\text {in}}\rangle = x$, we define $\varphi (x):= g(x)f(x)$ and equivalently search for fixed points satisfying $\varphi (x) = x$. Since $0 < \varphi (0)$, $\varphi (1) < 1$, and since *f*, *g*, and, hence, also $\varphi$ are all decreasing functions there is a unique root $\bar{x}_0$ in (0, 1), cf. Fig. 6. In conclusion,

#### Proposition 3.1

There is a unique stationary point $\bar{x}_0 \in (0,1)$ for the homogeneous problem (12). By extension this unique solution also applies to the homogeneous version of the full system (2).

Since we want to show that our system undergoes fate decision into a *non-homogeneous* solution, we next investigate the stability properties of the homogeneous steady state in the simplest one-dimensional setting consisting of two cells with a periodic boundary condition.

#### Proposition 3.2

The homogeneous stationary solution in the reduced system (3) is unstable in a system with two cells under a periodic boundary condition if and only if

13 $$\begin{aligned} f(\bar{x}_0)g'(\bar{x}_0)-f'(\bar{x}_0)g(\bar{x}_0)&< -1, \end{aligned}$$

for $\bar{x}_0$ the homogeneous stationary solution.

#### Proof

The two-cell periodic system reads

14 $$\begin{aligned} \left. \begin{aligned} \dot{x_1}&= g(x_2) f(x_1) - x_1 \\ \dot{x_2}&= g(x_1) f(x_2) - x_2 \end{aligned} \right\} . \end{aligned}$$

We assume small perturbations about the homogeneous steady state and introduce the change of variables

15 $$\begin{aligned} \begin{aligned} \sigma&= \frac{x_1 + x_2}{2}, \quad&\delta&= \frac{x_1 - x_2}{2}, \end{aligned} \end{aligned}$$

where we consider the perturbation $\delta$ small. Expanding the system around the homogeneous stationary solution, the equations decouple and we find the governing equation

16 $$\begin{aligned} \dot{\delta } = \left( f'(\sigma )g(\sigma )-f(\sigma )g'(\sigma ) -1 \right) \delta . \end{aligned}$$

Letting $\sigma = \bar{x}_0$ we obtain condition (13). $\square$

This result holds for parameter $(a,b) > 0$ which holds for all reductions to any of the reduced systems. That the previous result remains true for the full system (2) is more involved to show so we defer this to the next Sect. 3.2.

We next consider the existence of a non-homogeneous steady state. We again assume a 2-cell periodic set up and thus look for stationary solutions to (14).

#### Proposition 3.3

Under condition (13) there exists a non-homogeneous stationary state for the 2-cell periodic problem (14). By extension this solution also applies to the corresponding periodic version of (2).

#### Proof

From (14) we have the stationary relation

$$\begin{aligned} h(x_1):= \frac{x_1}{f(x_1)} = g(x_2), \hbox { or } x_1 = h^{-1}(g(x_2)) =: \gamma (x_2). \end{aligned}$$

For positive arguments, the function *h* is increasing, hence $h^{-1}$ is increasing too, and with *g* decreasing, $\gamma$ is therefore a decreasing function. One readily shows that $\gamma (0) > 0$ and $\gamma (1) < 1$ which together forms a second proof of the existence of the unique fix point $\bar{x}_0$ for the homogeneous stationary state. However, we are rather interested in cyclic solutions, i.e., for which $x = (\gamma \circ \gamma )(x) =: \gamma _2(x)$, since these correspond to alternating (patterned) solutions in the 2-cell problem. It is easy to see that $\gamma _2(0) > 0$ and $\gamma _2(1) < 1$ and since $\gamma _2(\bar{x}_0) = \bar{x}_0$ we find two additional solutions $\bar{x}_1< \bar{x}_0 < \bar{x}_2$ under the condition that $\gamma _2'(\bar{x}_0) > 1$, cf. Fig. 6 (*right*). We get

17 $$\begin{aligned} \frac{d}{d\xi } \gamma (\gamma (\xi )) \vert _{\xi =\bar{x}_0}> 1&\iff \gamma '(\gamma (\xi ))\gamma '(\xi ) \vert _{\xi =\bar{x}_0} = \gamma '(\bar{x}_0)^2 > 1 \iff \gamma '(\bar{x}_0) < -1. \end{aligned}$$

We find via implicit differentiation and using $\bar{x}_0 = \gamma (\bar{x}_0)$ that

$$\begin{aligned} \gamma '(\bar{x}_0)&= \frac{g'(\bar{x}_0)}{h'\left( \gamma (\bar{x}_0)\right) } = \frac{f(\bar{x}_0) g'(\bar{x}_0)}{1-\bar{x}_0 \, f'(\bar{x}_0)/f(\bar{x}_0)} = \frac{f(\bar{x}_0) g'(\bar{x}_0)}{1-g(\bar{x}_0)f'(\bar{x}_0)}, \end{aligned}$$

revealing that, in fact, (17) is equivalent to condition (13). $\square$

One cannot rule out the existence of more than one set of non-homogeneous solutions. To select a specific one, we pick the one pair $(\bar{x}_1,\bar{x}_2)$ which is the furthest away from $\bar{x}_0$. By inspection this solution also satisfies

18 $$\begin{aligned} \gamma _2'(\bar{x}_1) = \gamma _2'(\bar{x}_2) = \gamma '(\bar{x}_1) \gamma '(\bar{x}_2) < 1, \end{aligned}$$

cf. Fig. 6 (*right*). Interestingly, this property guarantees stability of this solution as we next demonstrate.

#### Proposition 3.4

The non-homogeneous solution of Proposition 3.3 is stable whenever it exists.

#### Proof

The Jacobian around the non-homogeneous solution has the characteristic polynomial

$$\begin{aligned} p(\lambda )&= (\lambda - f'_1g_2+1)(\lambda - f'_2g_1+1)- f_1 f_2 g_1'g_2', \end{aligned}$$

where $f_1 = f(\bar{x}_1)$ and similarly for $f'_1$, $g_1$, $g'_2$, etc. By inspection all coefficients are positive except for the 0th order term. By Descarte’s rule of sign there is a positive real eigenvalue if an only if this term is negative, that is, the non-homogeneous stationary solution is stable if and only if

19 $$\begin{aligned} 0&< f'_1f'_2g_1g_2 - f_1f_2g_1'g_2' - f'_1g_2 - f'_2g_1 + 1. \end{aligned}$$

For the function $\gamma$ introduced in the proof of Proposition 3.3, we have

$$\begin{aligned} \gamma '(\bar{x}_1)&= \frac{g'_1}{h'\left( \gamma (\bar{x}_1)\right) } = \frac{f_2 g'_1}{1-\bar{x}_2 \, f'_2/f_2} = \frac{f_2 g'_1}{1-f'_2g_1}, \end{aligned}$$

and similarly for $\gamma '(\bar{x}_2)$. Hence, from rearranging the property (18) we find

$$\begin{aligned} (1-f'_2g_1)(1-f'_1g_2)&> f_1 f_2 g'_1 g'_2, \end{aligned}$$

which is equivalent to condition (19). $\square$

**Fig. 7:**
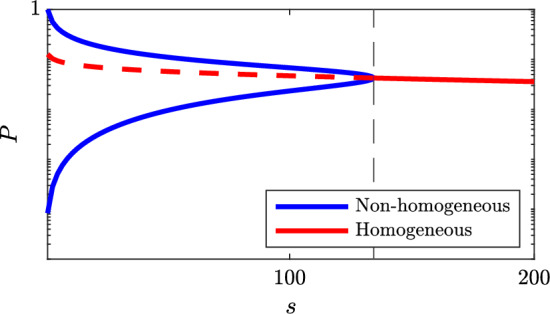
The non-homogeneous and homogeneous stationary states of the Hes1 protein *P* (log-scale), respectively, as a function of a scaling *s*, which acts upon the parameter $\alpha _N$, by scaling $\alpha _N \mapsto s^{-1}\alpha _N$. The homogeneous solution always exists, but is unstable to the left of the dashed line which indicates the smallest value of *s* for which (13) and (26) are true (color figure online)

So far we have shown that there always exists a unique homogeneous stationary solution. For the 2-cell periodic problem and under condition (13), this solution is unstable and there is then another non-homogeneous solution which *is* stable. Figure 7 illustrates this behaviour along a certain selected path in parameter space for the full model (2). We next proceed to show that as suggested by this graphic, the results indeed hold for the full model as well.

### Extension to the Full Model

To understand in what way the reduced models capture the stability properties of the full model, we need to describe how they are related at sufficient detail. Let a general ODE have the form $\dot{x} = F(x)$ and assume that the state has been split according to $x = [y; \; z]$, that is,

20 $$\begin{aligned} \begin{bmatrix}\dot{y} \\ \dot{z} \end{bmatrix}&= \begin{bmatrix} F_y(y,z)\\ F_z(y,z) \end{bmatrix}. \end{aligned}$$

The reduced model for *z* is obtained by assuming that $\dot{y} \approx 0$ and such that, given *z*, *y* can be uniquely solved for

21 $$\begin{aligned} 0&= F_y(y,z) \iff y = G(z). \end{aligned}$$

The reduced model is then simply

22 $$\begin{aligned} \dot{z}&= F_z(G(z),z), \end{aligned}$$

and the reduced model’s Jacobian is given by

23 $$\begin{aligned} J_z&= \partial _y F_z G'(z)+\partial _z F_z = -\partial _y F_z [\partial _y F_y]^{-1}\partial _zF_y+\partial _z F_z. \end{aligned}$$

By contrast, the full Jacobian reads

24 $$\begin{aligned} J&= \begin{bmatrix} \partial _y F_y & \partial _z F_y \\ \partial _y F_z & \partial _z F_z \end{bmatrix}, \end{aligned}$$

and by a block decomposition (Horn and Johnson 1999) the determinant is given by

25 $$\begin{aligned} \det (J)&= \det (\partial _y F_y ) \times \det \left( \partial _z F_z-\partial _y F_z [\partial _y F_y]^{-1}\partial _zF_y \right) . \end{aligned}$$

In general, both Jacobians *J* and $J_z$ depend on a parameter vector $\theta$, say, such that we can write $J = J(\theta )$ and equivalently for $J_z$. Since the determinant of the negative Jacobian is the 0th order term of the characteristic polynomial, we formulate the following lemma by comparing (23) and (25):

#### Lemma 3.5

Let $p_x(\lambda ) \equiv \det (\lambda I-J)$ be the characteristic polynomial for the full Jacobian and equivalently define $p_z(\lambda ) \equiv \det (\lambda I-J_z)$. Suppose that for some parameter $\theta$, all coefficients are positive except for possibly the 0th order term $p_x(0)$. Suppose also that the order reduction is definite in the sense that $\partial _y F_y$ is either positive or negative definite for all considered parameters $\theta$. Then, as a function of $\theta$, $p_x(0)$ switches sign simultaneously with $p_z(0)$ and in fact, $p_x(0) = \det (-\partial _y F_y) \times p_z(0)$.

The main use of the lemma is in conjunction with Descarte’s rule of sign as it allows one to conclude that the spectrum of *J* switches from stable to unstable at points for which $J_z$ is singular. The expressions for these points are typically simpler to obtain than for the full system. However, one still has to show that the full characteristic polynomial has positive terms of higher order than 0.

#### Proposition 3.6

Let $P_0$ be the homogeneous stationary solution for state *P* of the full model (2). This solution is unstable for the 2-cell periodic problem if and only if

26 $$\begin{aligned} -\frac{h (P_0/K_n)^h}{1+(P_0/K_n)^h}+ k P_0 (P_0/K_M)^k (1+(P_0/K_n)^h) \times R&< -1, \end{aligned}$$

where $$R \equiv \prod _i \alpha _i/\mu _i$$, $$i \in \{ D,N,M,P,n\}$$.

Under the reduction (3) (cf. Appendix B) we have that

27 $$\begin{aligned} a&= (K_M/R)^{k/(k+1)}, \end{aligned}$$

28 $$\begin{aligned} b&= \left( K_M^{k/(k + 1)}/K_n \times R^{1/(k+1)} \right) ^h, \end{aligned}$$

29 $$\begin{aligned} P_0&= R^{1/(k+1)} \times (\alpha _P/\mu _P)^{2k/(k+1)} K_M^{-k/(k+1)} \times \bar{x}_0, \end{aligned}$$

where we recall that $\bar{x}_0 \in (0,1)$ is the homogeneous stationary solution for the reduced model as in Proposition 3.1.

#### Proof

After the same type of change of variables as in (15) and linearising around small perturbations, we obtain a relatively sparse linear time-dependent system. The characteristic polynomial $p(\lambda )$ can therefore be obtained via iterated cofactor expansions. Writing $f_k(x) = 1/(1+x^k)$ and similarly for $f_h$, we find

$$\begin{aligned} p(\lambda )&= (\lambda + \mu _D)(\lambda + \mu _N)(\lambda + \mu _n) \left[ (\lambda + \mu _M)(\lambda + \mu _P) - N_0 \alpha _M \alpha _P f'_k(P_0/K_M)/K_M\right] \\&+\alpha _D \alpha _N \alpha _M \alpha _P \alpha _n f'_h(P_0/K_n) f_k(P_0/K_M)/K_n. \end{aligned}$$

By inspection all coefficients of the polynomial are positive except for possibly the constant term. We verify that $\det (-\partial _y F_y) = \mu _N \mu _M \mu _D \mu _n > 0$ in the notation of Lemma 3.5 and so it follows that the stability condition (13) is preserved by the state reduction. Using the relations (27)–(29) and the fix point relation $\bar{x}_0 = f(\bar{x}_0)g(\bar{x}_0)$ we find that (13) is equivalent to (26). $\square$

It remains to show that the non-homogeneous solution also shares its stability properties with the reduced model.

#### Proposition 3.7

The non-homogeneous solution of Proposition 3.3 is stable for the full model (2) whenever it exists.

#### Proof

This time we linearise around the non-homogeneous solution and obtain a 10-by-10 Jacobian. Luckily the Jacobian is rather sparse such that its characteristic polynomial can be expanded into

$$\begin{aligned} p(\lambda )&= (\lambda + \mu _D)^2(\lambda + \mu _N)^2(\lambda + \mu _n)^2 \times \\&\quad \phantom {=} \left[ (\lambda + \mu _M)(\lambda + \mu _P) - N_1\alpha _M\alpha _Pf'_k(P_1/K_M)/K_M\right] \times \\&\quad \phantom {=} \left[ (\lambda + \mu _M)(\lambda + \mu _P) - N_2\alpha _M\alpha _Pf'_k(P_2/K_M)/K_M \right] \\&\quad \phantom {=} - (\alpha _D\alpha _N\alpha _M\alpha _P\alpha _n/K_n)^2 f'_h(P_1/K_n)f'_h(P_2/K_n)f_k(P_1/K_M)f_k(P_2/K_M). \end{aligned}$$

All coefficients of the polynomial are positive except for possibly the constant term. The reduction map is verified to be positive definite and so we conclude that the stability condition (26) again controls the stability also of the non-homogeneous solution. $\square$

### Patterning on Regular Hexagonal Tilings

Next we are interested in analysing the patterning that occurs when a non-homogeneous steady state is reached. From a biological perspective, a “random” pattern or a chaotic non-stationary behaviour is implausible in a highly regulated pathway. As we have shown in Propositions 3.2 and 3.6, the homogeneous steady state is unstable in both the reduced and the full models under conditions (13) and (26), assuming a two-cell system with periodic couplings. When the homogeneous steady state *is* unstable, the heterogeneous steady state exists and is stable (Propositions 3.3, 3.4, 3.7). Again, this holds for the simple case of a two-cell system with periodic couplings, but we are nevertheless led to believe that a regular periodic pattern will eventually result from the model.

**Fig. 8:**
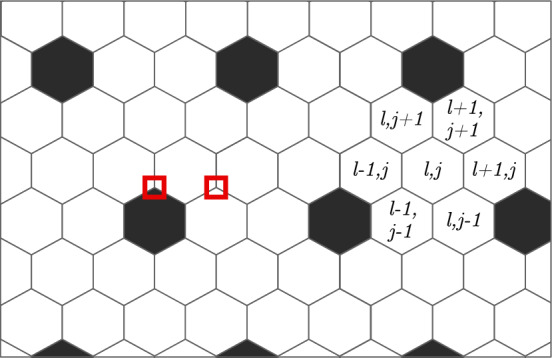
A regularly periodic pattern on a hexagonal grid with period 3 in each lattice direction. The labelling scheme shown follows (Collier et al. 1996). The red squares highlight two vertices which make the pattern non-transitive: the left borders 2 white and one black cell, while the right borders 3 white cells

**Fig. 9:**
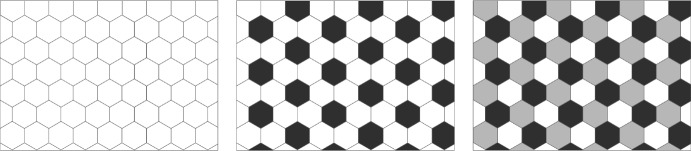
The three patterns on a regular hexagonal tiling which show vertex transitive behaviour, i.e., every vertex has the same neighbours. Note that the right pattern is identical to the middle pattern if two colours are the same

On a regular hexagonal tiling, there are a multitude of regularly periodic patterns that can occur, yet not every such periodic pattern is *uniform* or *vertex-transitive*. In the case of the non-uniform pattern illustrated in Fig. 8, for example, we require two ‘sub-types’ of white cells: those that border black cells and those that do not. We argue that for symmetry reasons, assuming a vertex-transitive pattern is a reasonable assumption for further investigations of our model. There exist three such uniform colourings on a regular hexagonal tiling (Grünbaum and Shephard 1987), see Fig. 9, one of which describes the homogeneous case.

#### Proposition 3.8

Under periodic boundary conditions the homogeneous steady state of the reduced system (5) is conditionally unstable in both one and two dimensions. Conditions for instability are the previous (13), while in 2D the condition is found in (30) below.

#### Proof

On a one-dimensional lattice with $j = 1,2,\ldots ,N$, we find from (5) the governing equations

$$\begin{aligned} \dot{x}_j&= \frac{g(x_{j-1})+g(x_{j+1})}{2} f(x_j)-x_j. \\ \end{aligned}$$

Linearising around the homogeneous solution and writing $x_j = \bar{x}_0+\delta _j$ we find

$$\begin{aligned} \dot{\delta }_j&= g'f \frac{\delta _{j-1}+\delta _{j+1}}{2} +gf' \delta _j-\delta _j. \end{aligned}$$

Inserting the Fourier representation

$$\begin{aligned} \delta _j&= \sum _{s=1}^N \xi _s \exp \left( \frac{2 \pi i s j }{N} \right) , \\ \end{aligned}$$

we get

$$\begin{aligned} \dot{\xi }_s&= \left( g'f\cos \left( \frac{2\pi }{N} s \right) +gf'-1\right) \xi _s. \end{aligned}$$

By inspection the most unstable case occurs for $s/N = 1/2$ which is then equivalent to condition (13).

On a two-dimensional, regular hexagonal lattice with $j=1,2,\ldots ,M$ and $l=1,2,\ldots ,N$, we similarly find the governing equations

$$\begin{aligned} \dot{x}_{lj}&= \frac{\sum g(x_{l\pm 1,j\pm 1})}{6} f(x_{lj})-x_{lj}, \end{aligned}$$

where the sum involves the 6 lattice neighbours, cf. Fig. 8. Linearising around the homogeneous solution, we find

$$\begin{aligned} \dot{\delta }_{lj}&= g'f \frac{\sum \delta _{l\pm 1,j\pm 1}}{6} +gf' \delta _{lj}-\delta _{lj}. \end{aligned}$$

Again making use of the Fourier representation,

$$\begin{aligned} \delta _{lj}&= \sum _{r=1}^M \sum _{s=1}^N \xi _{rs} \exp \left( \frac{2 \pi irl}{M} + \frac{2 \pi isj}{N} \right) , \end{aligned}$$

this becomes

$$\begin{aligned} \dot{\xi }_{rs}&= \left( g'f A+ gf'-1\right) \xi _{rs}, \end{aligned}$$

where

$$\begin{aligned} 3A&\equiv \cos \left( \frac{2\pi }{N} s \right) + \cos \left( \frac{2\pi }{M} r \right) + \cos \left( \frac{2\pi }{N} s + \frac{2\pi }{M} r \right) . \end{aligned}$$

The most unstable case occurs for $s = r = N/3$, assuming $N = M$ and divisibility by 3. The implied condition for instability is then

30 $$\begin{aligned} f(\bar{x}_0)g'(\bar{x}_0)/2-f'(\bar{x}_0)g(\bar{x}_0)&< -1. \end{aligned}$$

$$\square $$

It is tempting to draw the conclusion that the corresponding unstable frequency is also the resulting pattern: with period *N*/3 this would indeed imply the middle pattern in Fig. 9 which is also what we observe from numerical experiments. However, the analysis only reveals the most unstable modes around the homogeneous solution and does not predict the eventual end-fate.

From numerical experiments we consistently find that the typical stationary pattern generally matches that of Fig. 9 (*middle*), with black/white corresponding to, respectively, low/high Hes1 protein concentrations. Since the stationary state on the hexagonal mesh only consists of two distinct states it seems intuitive to attempt to analyse the two-dimensional situation by looking at the two-cell model coupled according to

31 $$\begin{aligned} W_2&= \begin{bmatrix} 0 & 1 \\ 1/2 & 1/2 \end{bmatrix}, \end{aligned}$$

i.e., as observed each black cell only has white neighbours while white cells have on average three white and three black neighbours. Thus, we consider the generic coupled model

32 $$\begin{aligned} \dot{x}_i&= \sum _j W_{ij} g(x_j) \times f(x_i)-x_i, \end{aligned}$$

for $i = 1,2$ and $W = W_2$. However, this immediate two-dimensional extension of the two-cell periodic one-dimensional case gives incorrect results. This case supports non-homogeneous *stable* solutions which are close to the homogeneous one but which are never observed in larger simulations.

**Fig. 10:**
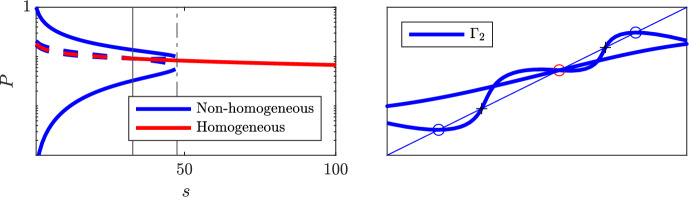
Stationary solutions under vanishing feedback. *Left:* bifurcation diagram of the Hes1 protein *P* as a function of scaling *s*; $\alpha _N \mapsto s^{-1}\alpha _N$. Below the boundary point defined by (36) (*dash-dot vertical*) there exist two pairs of non-homogeneous solutions, but only one pair is stable. Below the critical value (30) (*solid vertical*) the homogeneous solution is also unstable. *Solid/dashed* indicates stable/unstable solutions, respectively. *Right:* illustration of the fix point problem (35) when the parameter *s* is just above and below the limit for existence of non-homogeneous stationary solutions. When *s* increases, the pair of unstable solutions (*plus-signs*) approaches the pair of stable solutions (*circles*) until they collapse into a double root (condition (36)), after which only the homogeneous solution remains (color figure online)

A better generalisation of the observed behaviour on the hexagonal mesh is rather *three* cells, that is, the smallest integer multiple of three as suggested by the previous Fourier analysis. Namely, we take the generic model (32) with $W = W_3$,

33 $$\begin{aligned} W_3&= \begin{bmatrix} 0 & 1/2 & 1/2 \\ 1/2 & 0 & 1/2 \\ 1/2 & 1/2 & 0 \end{bmatrix}, \end{aligned}$$

and $i = 1,\ldots ,3$ to represent the vertex-transitive model matching Fig. 9 (*right*). Consider first the labels “low/medium/high” concentrations, say, at a stationary state $[x_1,x_2,x_3]$. Since the non-homogeneous stationary solution consists of either low or high concentration we will make the identification that “medium” corresponds to “high” concentration, i.e., $x_2 = x_3$, mimicking the way Fig. 9 (*right*) can be transformed into Fig. 9 (*middle*). Conveniently, the stationary states can now be found by considering the simpler extension (31)–(32) since the stationary relations are the same. Following the approach in the proof of Proposition 3.3 we have

34 $$\begin{aligned} \left. \begin{aligned} x_1&= g(x_2)f(x_1) \\ x_2&= \frac{g(x_1)+g(x_2)}{2} f(x_2) \end{aligned} \right\} \quad \left. \begin{aligned} h(x_1)&= g(x_2) \\ H(x_2)&= \frac{g(x_1)}{2} \end{aligned} \right\} \quad \left. \begin{aligned} x_1&= \gamma (x_2) \\ x_2&= \Gamma (x_1) \end{aligned} \right\} , \end{aligned}$$

where *H* and $\Gamma$ are defined in analogy with *h* and $\gamma$. Alternating (cyclic) solutions are now found from

35 $$\begin{aligned} x_1&= (\gamma \circ \Gamma )(x_1) =: \Gamma _2(x_1), \end{aligned}$$

and as before a sufficient condition for existence would be $\Gamma _2'(\bar{x}_0) > 1$. Unfortunately this approach fails due to the existence of multiple cyclic solutions as the numerical experiment in Fig. 10 explains. Except for in singular points there are now *two* pairs of non-homogeneous solutions and the crossing at the homogeneous solution generally satisfies $\Gamma _2'(\bar{x}_0) < 1$. Inspired by this graphical motivation, we instead proceed by assuming that non-homogeneous solutions exist for *some* parameter combination and we attempt to find points for which all such non-homogeneous solutions vanish.

#### Proposition 3.9

The boundary for existence of non-homogeneous solutions is defined by

36 $$\begin{aligned} \frac{g'_1f_2}{2-[f'_2g_2+f_2g'_2]-g_1f'_2} \times \frac{f_1g'_2}{1-f'_1g_2}&= 1, \end{aligned}$$

where $f_1 = f(\bar{x}_1)$ and similarly for $f'_1$, $g_1$, $g'_2$, etc.

#### Proof

By the graphical motivation in Fig. 10 *(right)* we search for a double root at the non-homogenous solution. That is, for which $\Gamma _2'(\bar{x}_1) = \Gamma '(\bar{x}_1)\gamma '(\bar{x}_2) = 1$. We find the derivatives through implicit differentiation leading to the two factors in the expression (36). $\square$

**Fig. 11:**
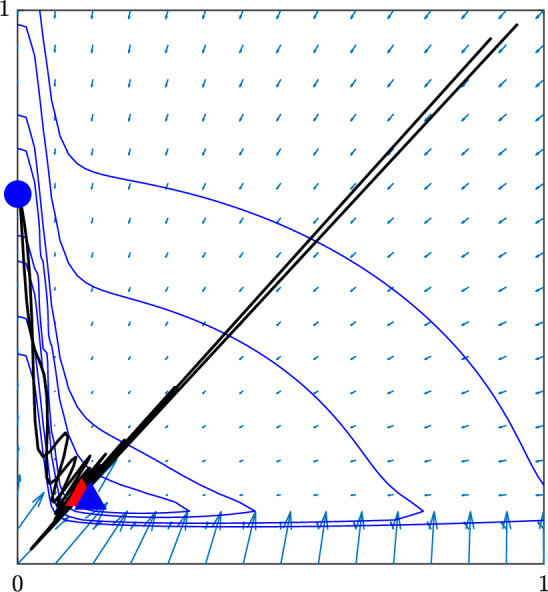
Phase-plot of the three-cell problem in the plane which contains all three stationary points. *Circle:* stable non-homogeneous solution, *triangles:* unstable solutions with red the homogeneous one. *Level curves* according to the Euclidean norm of the right-hand side and *arrows* denote the direction of the flow. Two sample trajectories starting from the upper right corner are also shown, with the oscillations visible before the fate decision (color figure online)

To sum up, under the proposed parameters from Table  1 and under weakened feedback $\alpha _N \rightarrow 0+$, the model undergoes a transition where the typical checkerboard patterning is lost. The two-dimensional generalisation into three cells, as given by (32)–(33), displays the same stability of post fate decision patterning as consistently observed for the full model when simulated over a grid of multiple connected cells. As an illustration, the overall typical dynamics of the three-cell system is summarised in Fig. 11

### System’s Size Convergence

**Fig. 12:**
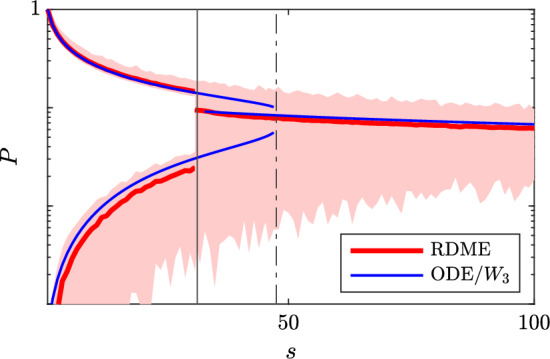
Same scaling as in Fig. 10, but instead solving the RDME-model on a two-dimensional hex-grid and estimating the mean behaviour of high and low protein concentrations among the cells. The stable solutions of the ODE for the three cell problem from Fig. 10 are included for reference (color figure online)

Finally we want to at least qualitatively investigate the RDME model (9)–(11). As with the ODE model, we first investigate its stability to scaling in the single parameter $\alpha _N$. Unlike the ODE model, one is now forced to use a statistical procedure to estimate when the non-homogeneous solutions are lost. This is complicated by the fact that the level of noise is rather large around the transition points, We find in Fig. 12 that while the bifurcation behaviour is not as clear as in Fig. 10, there is a gradual change of system behaviour approximately around the critical value(s) of scaling *s*, where the system behaviour changes from non-homogeneous (patterned) into homogeneous. In Fig. 12 we select the top 25% and bottom 25%, respectively, of the stationary Hes1 protein levels, and plot their means. We use the diameter of each sample as a measure of the spread and judge if the distribution is bimodal or not. Other statistical procedures yield slightly varying results but overall, we find that the RDME behaviour over a mesh is fairly well predicted by the solutions and critical points of the three cell problem.

**Fig. 13:**
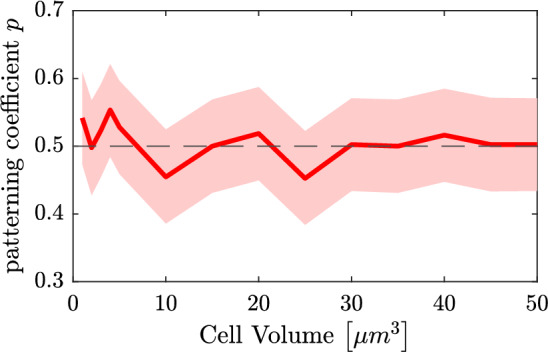
A measure of connectivity between high and low protein cells (perfect patterning corresponds to patterning coefficient $p = 1/2$) for a sequence of volumes in the RDME model (9). The numerical problem is treated as a statistical estimation problem for which the confidence intervals are indicated as a shaded area. See text for details (color figure online)

Finally, we try to measure the quality of the fate decision in the RDME model. For this purpose, recall the coupling matrix $W_2(i,j)$ as defined in (31), where *i*, *j* are the two different fates low/high Hes1 protein levels, respectively. For a perfect pattern with two final expression modes (Fig. 9 (*middle*)), the coupling $W_2$ is as given in (31). To evaluate the behaviour of the RDME model, we seek to estimate the effective coupling matrix from observations. By running multiple independent simulations and splitting the cells of the resulting stationary process into low/high Hes1 expression, we can count how often the specific coupling high-high occurs out of all possible couplings, that is, which corresponds to $W_2(2,2)$, the “patterning coefficient” *p*. We next treat these counts as independent Bernoulli trials and hence the set-up can be practically approached as a statistical estimation problem for the single Bernoulli parameter *p*. The results are summarised in Fig. 13 and indicate that even at relatively large levels of noise (corresponding to small cell volume), the patterning is quite close to the perfect one. For example, for mouse embryonal stem cells with a size of about $50\,\upmu$m$^3$, the GRN patterning behaviour is comparably stable to intrinsic cellular noise, with the patterning coefficient $\hat{p} = 0.50 (0.43,0.57)$ (95% CI).

## Discussion

Ultimately, we developed a first principle ODE model of the Hes1 pathway and its direct interactions with the Notch pathway, capturing oscillations followed by cell differentiation. We chose parameters based on biological data as much as feasible. By reducing our initial ODE model to lower dimensions, we were able to analyse the differentiation process. Furthermore, we extended the ODE model into a spatial stochastic RDME model to investigate the system’s robustness to intrinsic noise. We found that the transient oscillatory and the differentiation processes observed in the ODE model were well preserved in the RDME model. In this way we have found multiple interlinked ways to model this signalling process, allowing us to investigate multiple aspects of the behaviour of the Hes1-Notch pathway. Linking modelling frameworks in this way forced us to think hard about parameter scaling and parameterisation issues, particularly so in relation to the scarce availability of quantitative experimental data.

Our models capture essential aspects of the Hes1-Notch pathway, although they do not replicate every observed behaviour. We observed a few dampened oscillations followed by stable patterning both in the presence and absence of noise. However, the number of oscillations, their stability as well as their length is limited by parameter choice and the interpretation of the states included in the model. As stated in Kobayashi and Kageyama (2014), “[t]he molecular mechanism by which cells exhibit different (oscillatory vs. sustained) expression modes of Hes1 is still unknown”. Nevertheless, our results suggest that these distinct expression modes may be intrinsic to the GRN, and thus independent of external regulation.

Despite these promising findings, our models have some limitations. Our model’s oscillations, for example, are longer and more dampened than found in experimental data (Hirata et al. 2002; Imayoshi et al. 2013; Marinopoulou et al. 2021). However, more recent results suggest greater variability in oscillation periods in neural precursor cells with periods between 2–4 h (el Azhar et al. 2024) a range consistent with our results. Additionally, the final patterning behaviour of the RDME model (cf. Figs. 12 and 13) remains largely stable under noise, closely resembling the deterministic case. This suggests that noise does not crucially affect patterning behaviour in agreement with previous findings that stochastic behaviour in the Hes1 signalling pathway is stable to noise (Phillips et al. 2016). Such stability is desirable to such a developmentally significant differentiation pathway. While both the ODE and RDME behaviour (cf. Figs. 3 and 5) show similarities in oscillatory and fate decision behaviours, noise in the stochastic model allows for earlier fate decision in individual cells. As a result, oscillations appear less pronounced at the population level, again consistent with previous findings showing earlier differentiation in smaller systems (Phillips et al. 2016).

Our focus was on the Hes1-Notch pathway isolated from other cellular and signalling processes though we have made simplifications. We did not account for processes such as the dimerisation of the Hes1 protein before it induces mRNA production (Kageyama et al. 2007), its interactions with the micro RNA miR-9 (Goodfellow et al. 2014; Phillips et al. 2016) or other interactions with Notch effectors such as Mash1 (Shimojo et al. 2011). Additionally, the Hes1 pathway interacts with multiple other pathways, such as the cell cycle (Pfeuty 2015), RBP-J and Jagged (Kageyama et al. 2007), as well as the JAK-STAT pathway (Kobayashi and Kageyama 2014). These interactions could potentially stabilise the oscillations, which are currently severely dampened in our model. Furthermore, the addition of, for example, extra states or a delay to simulate this behaviour in an ODE system has been shown to allow for more stable oscillations (Goodwin 1965; Griffith 1968; Monk 2003; Jensen et al. 2003; Zeiser et al. 2007). However, such an extension of our model would further complicate mathematical analysis, which conflicts with our aim of balancing analysability and model complexity.

A major challenge in modelling cellular signalling pathways is the limited availability of quantitative data, leading to many models relying heavily or even exclusively on ad hoc parameter values chosen to fit expected model behaviour (Gunawardena 2010). As the aim of many Hes1 and Delta-Notch models is to hypothesise about how specific molecular interactions induce cellular and population-level behaviour, the significance of results not based on biologically relevant parameters have to be considered with caution. While qualitative time series data is more widely available, e.g., el Azhar et al. (2024), Imayoshi et al. (2013), fairly advanced parameter inference is needed to make effective use of this type of data, adding significant complexity. In our work, this data sparsity has particularly affected the parameters $\alpha _i, K_M, K_n, k$ and *h*. Nevertheless, we have scaled our model behaviour, and thus our parameters, to align with expected biological concentrations for the different constituents to improve the significance of our results. Since our primary focus is on model development and analysis, we leave more advanced parameter inference for future work. As a point in favour of our approach, the corresponding RDME model successfully simulates the Hes1-pathway down to the resolution of single species.

There are several promising ways to extend the work presented here. Extending the model to incorporate interactions with additional pathways, such as the JAK-STAT pathway or others, could further clarify the dynamics of Hes1 expression. Similarly, adding detail to the Hes1-Notch pathway itself to, for example, replace the phenomenological Hill functions with mechanistic models of gene regulation could improve model behaviour and give further insights into its oscillatory and sustained expression modes. Moreover, as the change between these two modes of expression happens during embryonal development to allow for sufficient numbers of neurons and glial cells to develop (Kobayashi and Kageyama 2014), considering the stability of this signalling process in a growing population becomes exceedingly relevant. Additionally, Hes1 is an important factor in the development of other tissues as well as different cancer types (Kobayashi and Kageyama 2014) so investigations of the differences between Hes1 interactions in these different tissues could be of interest as well.

To sum up, we have used different modelling approaches to capture the essential behaviours of the Hes1-Notch signalling pathway during neuronal development. By balancing simplicity and analytical tractability, we have constructed models that are both amenable to mathematical analysis and biologically meaningful. Using these models we capture the intrinsic expression of both oscillations and final patterning of this pathway while basing parameters on experimental data as much as possible. While there are improvements or different modelling emphases that can be chosen, our work promotes further theoretical understanding of the oscillatory and differentiation processes of this critical signalling pathway and the trade-offs between analysability and feasibility of the computational modelling.

### Availability and Reproducibility

The computational results can be reproduced with release 1.4 of the URDME open-source simulation framework (Drawert et al. 2012), available for download at (www.urdme.org). Refer to the Hes1 directory and the associated README.md in the DLCM workflow.

## A Parameterisation

From Ho et al. (2018), we know that the average amount of Hes1 protein in a *S. cerevisiae* cell is 8104 molecules. Since the yeast cells are roughly similar in size to mouse embryonal stem cells, we assume a size of $50\,\upmu$m$^3$ for both the *S. cerevisiae* cells and the mouse embryonal stem cells. This gives a wanted Hes1 protein concentration of $0.269\,\upmu$M. For the concentrations of the remaining constituents, we use data from the PaxDB database (Huang et al. 2023) using the values for the integrated whole organism of the mouse for Dll1, Notch1 and Hes1 while we use the Ngn2 value for the integrated whole organism of humans as this value was not available for mice. Since the values in PaxDB are given in parts per million, we use them to determine the concentrations of the constituents relative to each other. The values used and their scalings relative to Hes1 protein are shown in Table  3. Finally, we use results from Yu et al. (2006) to scale the Hes1 mRNA concentration. The authors show that there are $4.2 \pm 0.5$ protein molecules per mRNA molecule which gives a Hes1 mRNA concentration of $0.0613\,\upmu$M.

Using this information, we are able to scale $\alpha _i, K_M$ and $K_n$ such that the mean stationary behaviour is equal to those wanted concentrations in Table  3. After experimenting with this set-up, we fixed the Hill-function dissociation constants $K_M$ and $K_n$ at suitable values such that $\alpha _i$ are now uniquely defined as a function of all the other parameters. To find the distributions and confidence intervals of the activation rates, we perturb all degradation rates $\mu _i$ assuming they are log-normally distributed with a $68\%$ confidence interval as described in Table  1. We also perturb the desired concentrations in Table  3, assuming an ad hoc level of $5\%$ relative uncertainty as well as them being log-normally distributed. This way, we find perturbed activation rates $\alpha _i$ by fitting model behaviour to our previous requirements. We then fit lognormal distributions to the thus sampled $\alpha _i$ to find their confidence intervals as given in Table  1.

Finally, the distributions for all parameters of the full ODE model in Table  1 induce distributions of the reduced parameters, which we again find by fitting lognormal distributions to the perturbed reduced parameters, cf. Table  2.

**Table 3:** Concentration information for the four protein constituents of the model from Huang et al. (2023) and scaled to fit relative to Hes1 protein concentration

Molecule	PaxDB value	Scaled concentration
Dll1	0.03 ppm [*M. musculus*]	$0.0135 \ \upmu$M
Notch1	2.2 ppm [*M. musculus*]	$0.925 \ \upmu$M
Hes1 mRNA	–	$0.0613 \ \upmu$M
Hes1 protein	0.639 ppm [*M. musculus*]	$0.269 \ \upmu$M
Ngn2	0.12 ppm [*H. sapiens*]	$0.0505 \ \upmu$M

## B Dimensional Reduction

To reduce our ODE model (2) down to two equations, we rely on quasi-steady state assumptions. To show a typical reduction, we choose the third alternative as shown in Table 2, i.e. a type 1 reduction. Reductions of type 2 and 3 work equivalently and give the same values for *a* and *b*. The substitutions in this case are

$$\begin{aligned} \quad M \leftrightarrow x, \quad D \leftrightarrow y. \end{aligned}$$

We assume that the following variables are approximately stationary,

$$\begin{aligned} \begin{aligned} \dot{N} \approx 0 \implies N&= \frac{\alpha _N}{\mu _N} \langle D_{\text {in}}\rangle ,\\ \dot{P} \approx 0 \implies P&= \frac{\alpha _P}{\mu _P} M, \\ \dot{n} \approx 0 \implies n&= \frac{\alpha _n}{\mu _n} \frac{1}{1+(P/K_n)^k}, \end{aligned} \end{aligned}$$

where $$\langle D_{\text {in}}\rangle := \sum _i w_i D_i$$.

This gives

$$\begin{aligned}&\left. \begin{array}{rcl} \dot{M} & =& c_1 \frac{\langle D_{\text {in}}\rangle }{1 + c_2 M^k} - \mu _M M \\ \dot{D} & =& c_3 \frac{1}{1 + c_4 M^h} - \mu _D D \end{array} \right\} \end{aligned}$$

with

$$\begin{aligned} c_1&= \frac{\alpha _N \alpha _M}{\mu _N}, \quad c_2 = \left( \frac{\alpha _P}{\mu _P K_M} \right) ^k, \quad c_3 = \frac{\alpha _D \alpha _n}{\mu _n}, \quad c_4 = \left( \frac{\alpha _P}{\mu _P K_n} \right) ^h. \end{aligned}$$

Using the scaling

$$\begin{aligned} x = \frac{M}{M_0}, \quad y = \frac{D}{D_0}, \quad \tilde{t} = \frac{t}{\tau }, \end{aligned}$$

we find the non-dimensionalised equations

$$\begin{aligned}&\left. \begin{array}{rcl} \dot{x} & =& \frac{\langle y_{\text {in}}\rangle }{a + x^k} - x \\ \dot{y} & =& v \left( \frac{1}{1 + b x^h} - y \right) \end{array} \right\} \end{aligned}$$

where

$$\begin{aligned} M_0 = \left( \frac{c_1 D_0}{\mu _M c_2} \right) ^{\frac{1}{k+1}}, \quad D_0 = \frac{c_3}{\mu _D}, \quad \tau = \frac{1}{\mu _M}, \end{aligned}$$

and

$$\begin{aligned} a^{-1} = c_2 M_0^k, \quad b = c_4 M_0^h, \quad v = \frac{\mu _D}{\mu _M}. \end{aligned}$$
